# Publisher Correction: Dramatic HIV DNA degradation associated with spontaneous HIV suppression and disease-free outcome in a young seropositive woman following her infection

**DOI:** 10.1038/s41598-020-70050-w

**Published:** 2020-09-04

**Authors:** Philippe Colson, Catherine Dhiver, Catherine Tamalet, Jeremy Delerce, Olga O. Glazunova, Maxime Gaudin, Anthony Levasseur, Didier Raoult

**Affiliations:** 1 IHU Méditerranée Infection, 19-21 Boulevard Jean Moulin, 13005 Marseille, France; 2 Aix-Marseille Univ., IRD, APHM, MEPHI, 19-21 boulevard Jean Moulin, 13005 Marseille, France

Correction to: *Scientific Reports* https://doi.org/10.1038/s41598-020-58969-6, published online 13 February 2020.

The original version of this Article contained an error in the title of the paper, where ‘Full-length title:’ was incorrectly included. This has now been corrected in the PDF and HTML versions of the Article and in the Supplementary Information File.

